# Decoding the Interplay between Topology and Surface Charge in Graphene Oxide Membranes During Humidity Induced Swelling

**DOI:** 10.1021/acsnano.3c08260

**Published:** 2023-11-02

**Authors:** Mohd Rafie bin Shaharudin, Christopher D. Williams, Amritroop Achari, Rahul R. Nair, Paola Carbone

**Affiliations:** † Department of Chemical Engineering, School of Engineering, The University of Manchester, Booth Street East, M13 9PL Manchester, United Kingdom; ‡ Division of Pharmacy and Optometry, School of Health Sciences, The University of Manchester, Oxford Road, M13 9PT Manchester, United Kingdom; § National Graphene Institute, The University of Manchester, Booth Street East, M13 9PL Manchester, United Kingdom

**Keywords:** Graphene oxide, Membrane, Morphology, Surface charge, Nanomaterials, Swelling, Adsorption

## Abstract

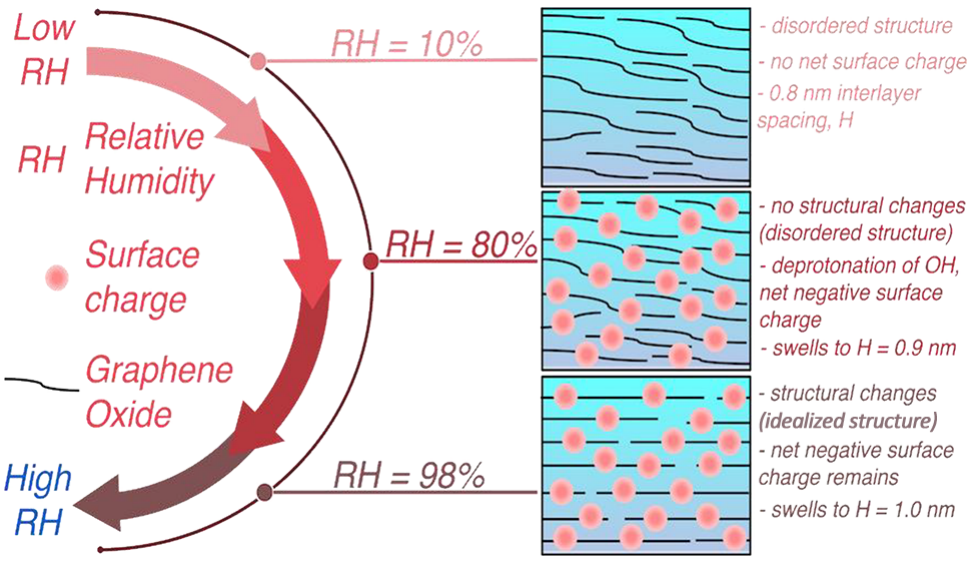

Graphene oxide (GO) membranes are known to have a complex morphology that depends on the degree of oxidation of the graphene flake and the membrane preparation technique. In this study, using Grand Canonical Monte Carlo simulations, we investigate the mechanism of swelling of GO membranes exposed to different relative humidity (RH) values and show how this is intimately related to the graphene surface chemistry. We show that the structure of the GO membrane changes while the membrane adsorbs water from the environment and that graphene oxide flakes become charged as the membrane is loaded with water and swells. A detailed comparison between simulation and experimental adsorption data reveals that the flake surface charge drives the water adsorption mechanism at low RH when the membrane topology is still disordered and the internal pores are small and asymmetric. As the membrane is exposed to higher RH (80%), the flake acquires more surface charge as more oxide groups deprotonate, and the pores grow in size, yet maintain their disordered geometry. Only for very high relative humidity (98%) does the membrane undergo structural changes. At this level of humidity, the pores in the membrane become slit-like but the flake surface charge remains constant. Our results unveil a very complex mechanism of swelling and show that a single molecular model cannot fully capture the ever-changing chemistry and morphology of the membrane as it swells. Our computational procedure provides the first atomically resolved insight into the GO membrane structure of experimental samples.

Recently a new type of carbon-based laminated membranes formed by stacks of graphene oxide (GO) flakes have emerged as a promising alternative for water purification processes.^1−5^ GO membranes swell in a humid environment and when immersed in water.^6−10^ Such expansion enables the formation of “permeation channels” whose size defines the permeation properties of the membranes.^11−14^ In a dry state, the size of the interlayer spacing, *H*, between GO flakes is around 0.8 ± 0.1 nm,^5−7^ making it small enough to reject the majority of large molecules and hydrated ions from permeating through while the negative surface charge on the GO flakes, due to ionization of functional groups, repels anions.^15^ When exposed to a humid environment, the expansion of the membrane occurs gradually with the size of *H*, increasing from 0.8 to 1.0 nm within a relative humidity (RH) range between 10 and 98%,^6,7^ while an abrupt increase of *H* to 1.3 nm is observed when the GO membrane is immersed in liquid water.^9^ The gradual swelling is attributed to the effect of intrastratification of water within the membrane layers that are partially hydrated with clusters of water around the hydrophilic regions containing oxygenated functional groups.^16,17^

Since the size of the permeation channels defines the selectivity of the GO membrane toward dissolved ions and other species,^11−13,18−21^ the swelling mechanism and its control is a key property enabling practical applications. Thus, control of swelling is paramount for the use of GO membranes for filtration applications. Experimental results have indicated that the swelling can be controlled mechanically and chemically.^22,23^ Abraham et al.^13^ showed that physical confinement using an epoxy resin encapsulant can prevent swelling of GO membranes, ensuring the selectivity of the membrane is retained during desalination. Zhao et al.^23^ have also shown that by thermally reducing the oxygen functional groups the degree of swelling of GO membranes is reduced as there are fewer oxygenated functional groups to act as spacers. Other attempts at controlling the swelling of GO membranes use linkers made of polymer,^24−26^ external pressure^22^ or cation linkers.^27^ However, from a macroscopic perspective, the root cause of the swelling cannot be easily pinpointed as both structural features of the membrane and the pH of the solution^15,28,29^ affect the experimental results. In particular, the former is far more complicated than the idealized picture routinely used to depict GO membranes. The complex topology of GO membranes has been widely reported in literature.^8,17,30−33^ Due to the presence of oxygenated functional groups, which modifies the surface chemistry and causes mechanical tension within the graphene sheets, the GO flakes become corrugated and wavy with protrusions and valleys. Moreover, during the assembly of GO membranes (usually performed by vacuum filtration), the random, irregular shapes and sizes of GO flakes lead to formation of offsets (i.e., gaps between flakes), curved and complex channel structure, and possibly overlapping edges. This complex internal morphology, difficult to characterize and control, coupled with a variable surface charge (depending on the degree of oxygenation^6,34,35^ and the solution pH^15,28,36^), is responsible for the lack of a reliable model to predict GO membrane swelling and currently hinders their practical use.

Molecular modeling can help in identifying and isolating the various effects at play when GO membranes swell as it allows for an investigation on specific aspects of the membrane including its hydrophilicity, which, as we will show, is not constant but varies depending on the relative humidity of the environment. This variability is caused by the presence on the GO flake of different types of oxygenated functional groups^37^ characterized by different p*K*_a_ values^38^ and therefore ionized at different stages of the swelling. In most computational studies, GO membrane models are however typically oversimplified with the channel modeled as a slit pore with predetermined *H* and no ionization of functional groups on the GO flake.^9,19,20,39,40^ Attempts at modeling a more realistic structure have been carried out by Gogoi et al.,^41,42^ who placed GO flakes in predetermined positions, leading to the formation of offsets that create a tortuous pathway for water, but the model is still not as complex as the real structure of GO membranes. A procedure for fabricating a more complex morphology of GO membranes was proposed by Williams et al.,^43^ who employed multistep molecular dynamics (MD) simulations to mimic the vacuum filtration preparation method of GO membranes. The procedure has been used by Reddy et al. to study the purification of pharmaceutical effluent, showing that GO membranes are promising candidates also for such filtration processes.^11^ Garcia et al. proposed a framework on producing a complex GO flake that could include defects and spatial distribution of oxygenated functional groups to capture the complexity of GO.^44^ Another method of fabricating the complex morphology of GO membrane models was also proposed by Chen et al. based on a pressure-assisted ultrafiltration self-assembly method. This model was then used to study the separation of heavy metal ions from water and the rejection mechanism.^45^ The results of these computational studies, along with the spread of the experimental data, clearly indicate that membranes with different morphologies perform differently.

This work aims to provide insight into the relationship between membrane morphology and humidity conditions as the membrane swells by exploring the effect of GO membrane morphology and surface charge on the adsorption of water and validating the model with experimental adsorption data. In our previous computational study,^46^ we showed that the inclusion of ionized functional groups in the model of the GO flakes is important for predicting the mechanical stability of the membrane as RH changes. Indeed, as various other studies indicated, the presence of water molecules in the membrane induces deprotonation of the oxygenated functional groups, leading to a net negative surface charge on the GO flakes.^36,38,47,48^ These ionized functional groups play a very important role in the swelling of GO membranes and can be the major cause of membrane delamination starting from low RH for two main reasons. First, at low RH values they initially act as adsorption sites for the water molecules,^46^ with which they interact via hydrogen bonding,^49^ and later, as the membrane fills with water, they become nucleation centers for further water adsorption. Second, their presence creates a strong electrostatic repulsion between the flakes.^36,47^ Thus, in addition to the solvation pressure induced by the accumulation of water in the small, confined space between the sheets,^50^ strong electrostatic repulsions overcome the attractive intersheet van der Waals (vdW) interactions, resulting in an increase in *H* or even delamination.

Undeniably, the contribution of ionized functional groups is very important and from our previous study, we have shown the importance of having a GO model incorporating surface charges and how the degree of ionization of the functional groups can be a guideline to control the mechanical stability of GO membranes.^46^ However, in comparison with experimental studies, our model shows an overprediction in the amount of water adsorbed at low humidity. Here we show that such overprediction is caused by the oversimplified slit-like structure of the pore, which makes the membrane model too hydrophilic at low relative humidity. The aim of the present work is to establish a rigorous relationship between surface charge, pore geometry, and relative humidity for GO membranes and provide an atomistic insight into the membrane structure as the membrane swells. To do that, we produce a calibration curve that correlates chemical potential, μ, to RH to allow us to directly compare our adsorption simulation results with experimental data.^6^ We then utilize the calibration curve to determine the value of μ based on the value of RH that is reported in the experimental literature for the water adsorption simulations of GO membranes. The adsorption simulations are carried out on two morphology types: one with an idealized pore geometry (i.e., slit-like) and one with a disordered structure (i.e., irregular pores). The results are then analyzed to determine which model is the best at reproducing experimental data at three humidity conditions and interlayer spacings. Discussions on the effect of morphology and surface charge that allow a specific model to be able reproduce experimental data are presented in terms of the amount of water adsorbed, the adsorption mechanism, favorable adsorption sites, and the free pore volume available within each membrane model.

## Results and Discussion

### Thermodynamics of Adsorption

**Figure 1:**
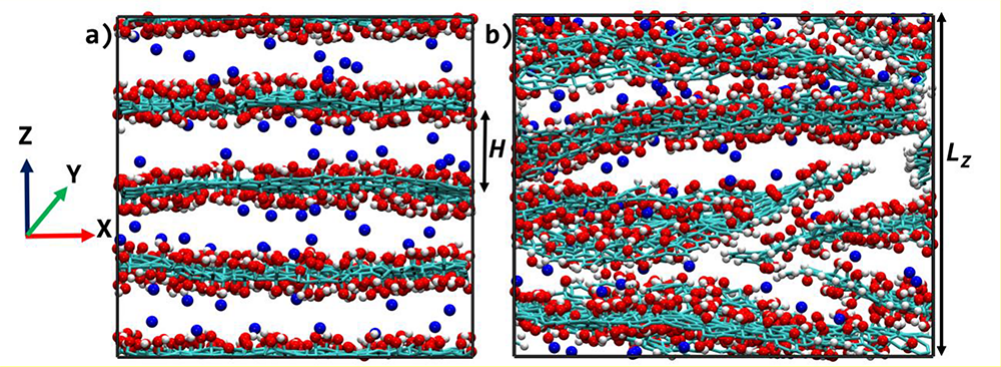
Initial configuration of (a) idealized and (b) disordered (right) GO membrane used in this study. Both models shown are of *H* = 1.0 nm and 10% deprotonation (10QGO) with water molecules omitted for clarity. Blue, sodium ion; red, oxygen atom; white, hydrogen atom; cyan, graphene backbone (carbon atom).

**Figure 2:**
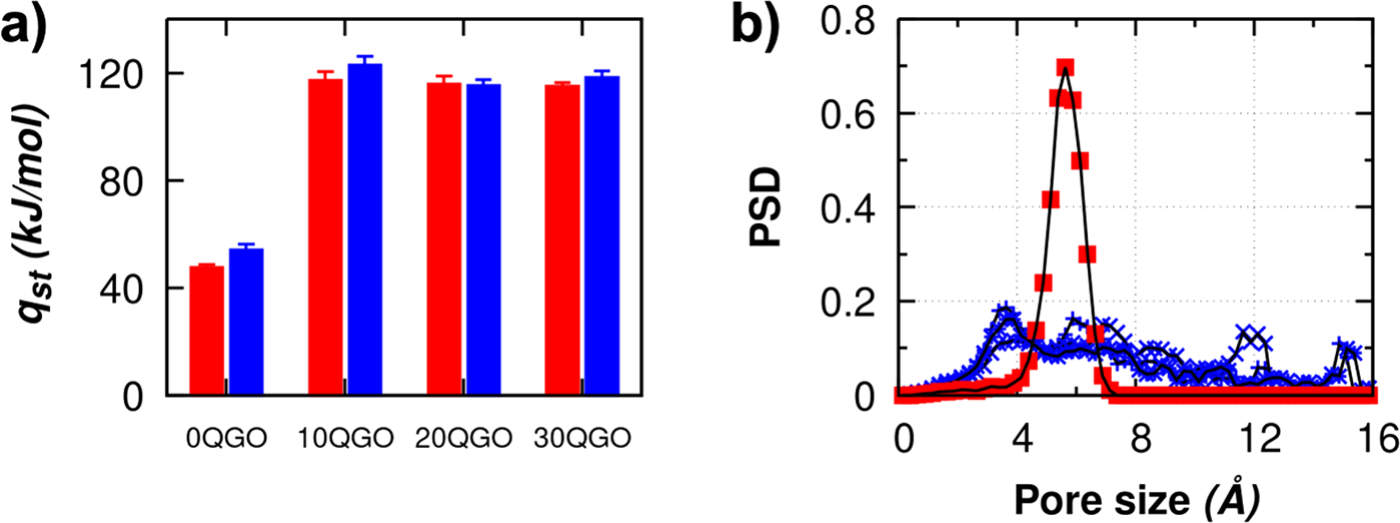
(a) Isosteric heat of adsorption (*q*_st_) in GO membrane at low loading for the disordered (blue) and idealized (red) model. (b) Pore size distribution (PSD) of the idealized (red filled squares) and disordered model 1 (blue plus signs), model 2 (blue times sign), and model 3 (blue asterisks) GO membrane. Three models of disordered membranes are fabricated to get the average. PSD shown here is for the *i*0QGO an*d*0QGO at *H* = 1.0 nm. PSD of the 10QGO, 20QGO, and 30QGO are provided in SI Figure S2.

To initially verify whether the topology of the membranes, idealized (see Figure 1a) or disordered (see Figure 1b), affects the thermodynamics of adsorption, we compare the isosteric heat, *q*_st_, for each model (Figure 2a) at low loading. *q*_st_ is calculated using the ensemble fluctuation approach (eq 1) suitable for nonzero loads.^51^ Since the calculation is made at low loading, the value of *q*_st_ calculated describes the strength of interaction between water molecules and the site of adsorption and it is calculated as
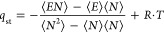
1where *E* is the total configurational energy of the system, *N* is the total number of water molecules adsorbed, *R* is the gas constant, *T* is the temperature of the system at 300 K, and the angular brackets denote ensemble averaging. The *q*_st_ value calculated from the ensemble fluctuation approach is the covariance of energy of adsorption of adsorbate divided by the variance of the number of adsorbates, which, for our system,^52^ describes the average strength of interaction between water molecules and the adsorption site. As both models have the same type of functional groups, both idealized and disordered membranes with net negative surface charge show a comparable *q*_st_ at all degrees of ionization and that the most favorable site for adsorption is the deprotonated hydroxyl (OH) group.

To confirm the preferred adsorption sites, we calculate the radial distribution function (RDF) between the center of mass distance, *r*, of hydrogen atoms of water molecules and the oxygen atom of the functional groups (Figure S1 in the Supporting Information (SI)). The RDF shows a strong peak at *r* = 0.175 nm,^53^ which is within the range of the geometrical definition of hydrogen bonding.^54^ This strong peak is not observed in the RDF between the water molecule hydrogen atoms and the oxygen atom of either epoxide (OE) or the protonated OH groups, suggesting that these are less likely to be the adsorption sites^55^ and further confirming that, at low loading, water molecules are most likely to be adsorbed at the ionized OH functional groups. The RDFs also explain why the isosteric heat calculated for the neutral membrane models, which lack ionized OH groups, is almost half that of the charged ones.

Most importantly, the isosteric heat values provide insight into how the topology of the membrane affects the thermodynamics of adsorption. Neutral GO models with disordered topology are characterized by slightly higher *q*_st_ value than the ordered ones. This result is the consequence of the models’ different pore size distributions (PSDs). The PSD, reported in Figure 2b, calculated using PoreBlazer version 4.0,^56^ shows that in *d*0QGO the PSD is wider with a high probability of finding smaller pore sizes (<0.3 nm) within the channel compared to the *i*0QGO that have a narrow PSD with a single sharp peak. In these small uncharged pores, the adsorption energy dominated by the attractive short-range van der Waals (vdW) interaction between water molecules and the atom of the GO flakes is stronger than in the larger ones due to the deeper energy well generated by the close proximity of the GO flakes forming the pore.^57,58^ Here we show that, at low loading and in a neutral state, the morphology of GO membranes has an effect on the thermodynamics of adsorption and the *d*0QGO is more hydrophilic than the *i*0QGO. The effect of the morphology, however, is not strong enough to have any significant effect in the charged GO models as the ionized OH functional groups dominate the adsorption process at low load.

### Amount of Water Adsorbed and Its Relation with the Membrane Topology and Surface Charge

**Figure 3:**
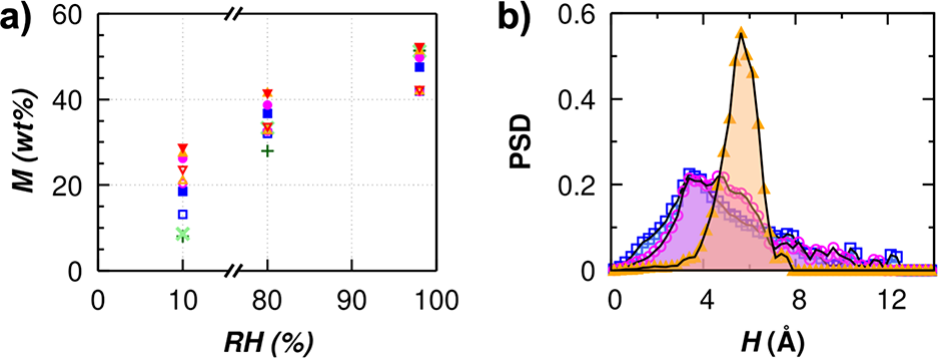
(a) Amount of water adsorbed for all GO models in this study compared to experimental data^6^ and (b) PSD for GO membranes that best predict experimental results at each RH condition (10, 80, and 98%). The PSD for disordered membrane is averaged over three models. Symbol definitions: +, Liu et al. experiment; ×, this work experiment; □, *d*0QGO; ■, *i*0QGO; ○, *d*10QGO; •, *i*10QGO; △, *d*20QGO; ▲, *i*20QGO; ▽, *d*30QGO; ▼, *i*30QGO.

The amount of water adsorbed, *M*, is expressed in the term of weight percentage (wt %) using eq 2;
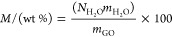
2where *N*_H_2_O_ is the number of water molecules adsorbed in GO membrane, *m*_H_2_O_ is the molar mass of one water molecule, and *m*_GO_ is the molar mass of the GO membrane. The calculations are repeated for each model at each interlayer distance, and the values reported in Figure 3a and Table 1 are one of each idealized model membrane and the average over three models for the disordered membrane. The computed data are reported along with the experimental values obtained by Liu et al.^6^ and our experimental data. The PSD of the best model reproducing experimental data is plotted in Figure 3b.

**Table 1:** Mass of Water Adsorbed, *M* (wt %), in GO Membrane with Interlayer Distance, *H*, Respectively, to the Relative Humidity (RH)^a^

	*M* (wt %)		
GO membrane model	RH (%) = 10; *H* (nm) = 0.8	RH (%) = 80; *H* (nm) = 0.9	RH (%) = 98; *H* (nm) = 1.0
*d*0QGO	13.13 ± 0.86	32.00 ± 0.24	41.90 ± 0.24
*d*10QGO	20.32 ± 0.63	32.58 ± 0.11	41.86 ± 0.28
*d*20QGO	21.14 ± 0.35	32.85 ± 0.18	42.04 ± 0.12
*d*30QGO	23.49 ± 0.31	33.53 ± 0.51	42.17 ± 0.03
*i*0QGO	18.46 ± 0.08	36.66 ± 0.03	47.54 ± 0.03
*i*10QGO	26.18 ± 0.03	38.73 ± 0.03	49.74 ± 0.04
*i*20QGO	27.63 ± 0.02	41.59 ± 0.02	51.49 ± 0.03
*i*30QGO	28.63 ± 0.03	41.35 ± 0.02	52.19 ± 0.02
Experiment			
this work	8.56	33.27	51.31
Liu et al.^6^	8.00	28.00	51.40

a Experimental data are from Liu et al.^6^ and our own adsorption experiments. The *M* of the GO model at RH = 100% is provided in SI Table S1.

For each RH, the water adsorption values predicted by the various models are quite different from each other. At low RH = 10%, the value of *M* is overpredicted by all GO membrane models but the model that provides the prediction by far closest to the experimental data is the disordered topology neutral membrane one (*d*0QGO). The value of *M* obtained with this model is still somewhat higher than the experimental data but significantly smaller than those obtained for the charged models and ordered ones. This result provides important information about the membrane topology and charge at such low partial pressure. At low RH, the amount of water in the membrane is small and not enough either to lead to the ionization of the OH groups or to build up enough hydration pressure to expand the pores. The overprediction of *M* in our model might be due to the assumption that in vacuum GO membranes are completely dry. Indeed, as proven experimentally by Yadav et al.^33^ through thermogravimetric analysis and via computer simulation by Williams et al.,^43^ the water content of GO membranes is never zero even at low relative humidity. Besides, due to the stochastic nature of Grand Canonical Monte Carlo (GCMC) simulation, there is also a possibility of water being inserted into pores that are not physically accessible in real adsorption processes due to GO sheets collapse.

**Figure 4:**
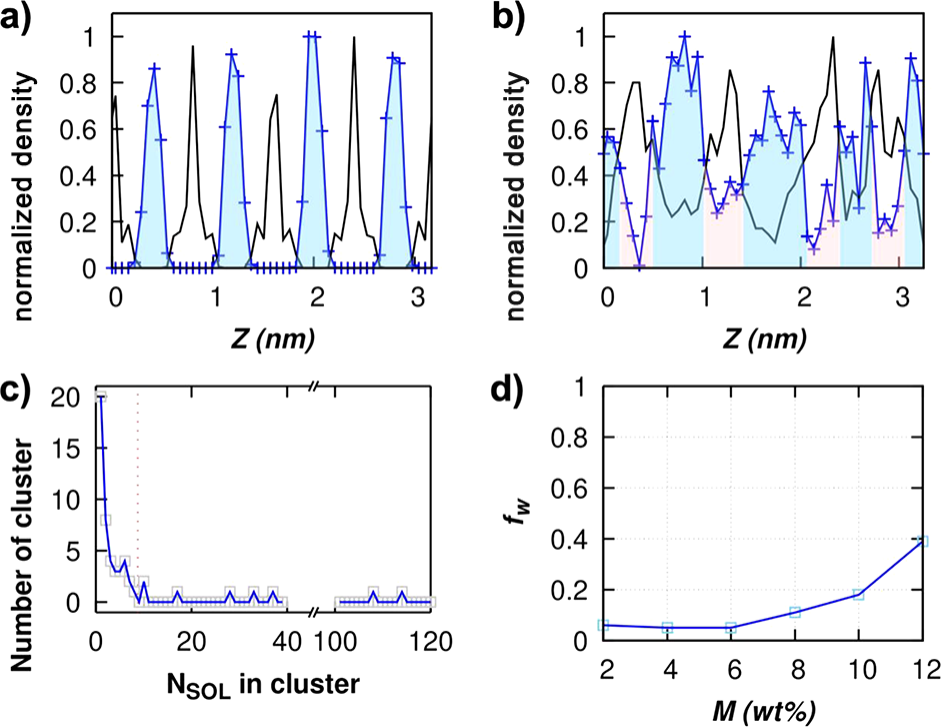
Density profile of water and GO flakes in the *Z* direction for (a) *i*0QGO and (b) *d*0QGO for one of the fabricated models, at RH = 10% and *H* = 0.8 nm (results of the other two models of *d*0QGO provided in SI Figure S3). The blue line indicates water, the black line indicates GO flake, blue shading indicates water adsorbed in the lamellar channels, and red shading indicates water adsorbed in small irregular pores. Water cluster distribution in (c) *d*0QGO model 1 (results of model 2 and model 3 provided in SI Figure S3), with water clusters below the mean cluster size (red dashed line) being attributed to water adsorbed in isolated pores, while the water cluster above the mean cluster size is attributed to water adsorbed in the lamellar region. (d) *f*_w_ of *d*0QGO up to the maximum water adsorbed at RH = 10%.

Looking back to the isosteric heat of adsorption in Figure 2, the neutral disordered model (*d*0QGO) has a slightly stronger adsorption energy than the neutral idealized one (*i*0QGO) due to the presence of smaller (and irregular) pores, hinting that more water should be adsorbed in the disordered membrane model than the idealized one. However, the small pores that have strong adsorption interaction with the water are not homogeneously distributed and some of them (those around the collapsing edges of the GO flakes) might be too small for water to be adsorbed. The idealized model on the other hand has a more homogeneous channel structure across the membrane, and the energetically favorable adsorption location from the vdW attraction of two opposing GO flakes is equally available at every channel within the membranes, providing a higher probability for water molecules to be adsorbed. The density profile of water adsorbed in *i*0QGO and *d*0QGO together with the water cluster distribution of Figure 4 show that water adsorption in *i*0QGO occurs within the lamellar channels with a comparable amount of water adsorbed at every layer, while *d*0QGO have water adsorbed in both the lamellar region and irregular isolated pores. The water cluster size distribution for *d*0QGO shows that only *M* = 3 wt % is adsorbed in the isolated pores, while the majority of water (*M* = 9 wt %) is adsorbed in the lamellar region with high pores interconnectivity. Our computer simulation result confirms the suggestion made by Yadav et al.^33^ that water molecules are mostly adsorbed within the lamellar region of the membrane at low RH before accommodating the more irregular pores (voids) at higher RH.

At RH = 80%, the idealized GO membrane models still overpredict the amount of water adsorbed when compared to experimental data, while the disordered models agree well with them. This indicates that, at this relatively high RH, the structure of the membrane is still disordered but has acquired a surface charge. The influence of the free pore volume in the disordered models shows a more profound effect to *M* than the idealized models. The difference in *M* between the disordered models (*d*0QGO – *d*30QGO) is minuscule. The effect of surface charge in the disordered models at an RH of 80% is less dominant compared to the topological influence on *M* as the disordered models are now saturated with water. The disordered GO membrane model that best predicts the experimental data at this RH is either *d*20QGO or *d*30QGO based on the value of *M*. Even though the *d*30QGO models have a better agreement with experimental *M*, this very high surface charge is not observed experimentally even when the GO membrane is immersed in liquid water. Therefore, we conclude that the best GO model in reproducing experimental data at this relatively high RH is the *d*20QGO model, which shows that, at RH = 80%, the membrane swells from 0.8 to 0.9 nm and acquires full surface charge while still maintaining the disordered topology.

**Figure 5:**
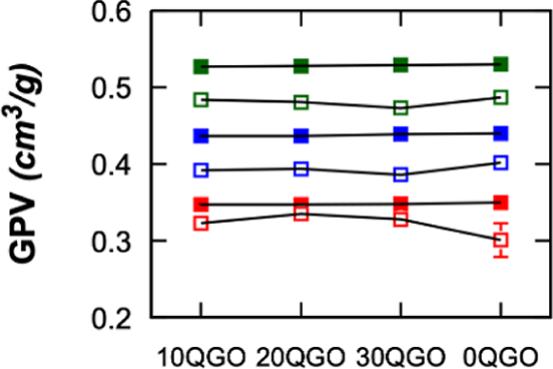
Geometric pore volume of idealized and disordered GO model at *H* sizes of 0.8 (red), 0.9 (blue), and 1.0 nm (green) calculated without the counterions. Filled markers are for idealized models, while open markers are for disordered models. Dashed lines are guides for eyes.

At high RH = 98%, the GO membrane model that best predicts experimental data is the idealized model with 20% ionization of functional groups, *i*20QGO. The effect of surface charge at high RH reduces as the adsorption near the saturation point is driven by water–water interaction, and the total capacity of the membrane is dictated by the free pore volume within the membrane. This observation at high humidity has been reported with more details in our previous work.^46^ The total amount of water that can be accommodated within the disordered GO model is lower than that of the idealized model since the geometrical pore volume in the former is always lower than in the latter at every *H* size, as shown in Figure 5. The difference between the geometric pore volume of the disordered and idealized models is about 10%, and this is reflected well in the amount of water adsorbed at high RH with the idealized model adsorbing about 10% more water compared to the disordered model. As seen at RH = 80%, the disordered models are already saturated with water molecules and are no longer a suitable model to represent the GO membrane topology as the membrane undergoes a structural transition from disordered to idealized, as more water molecules are adsorbed.

At this high RH approaching the saturation point, the idealized channel model best represents the structure of the GO membrane as the membrane has now swollen due to the solvation pressure created by the confined water layers and strong electrostatic repulsion from the surface charge. When the membrane is immersed in liquid water RH = 100% and *H* = 1.3 nm, the same trend is observed as at RH = 98% where the water adsorption capacity of the membrane is controlled by the pore volume of the membrane. The disordered models are strictly limited by the free pore volume within the membrane available to accommodate water even at high RH, and the presence of counterions does not show any influence on the amount of water adsorbed as the *d*10QGO, *d*20QGO, and *d*30QGO have a similar amount of water adsorbed of about ∼65 wt %. The *M* of all GO membranes at RH = 100% is provided in SI Table S1.

### Water Adsorption Mechanism

**Figure 6:**
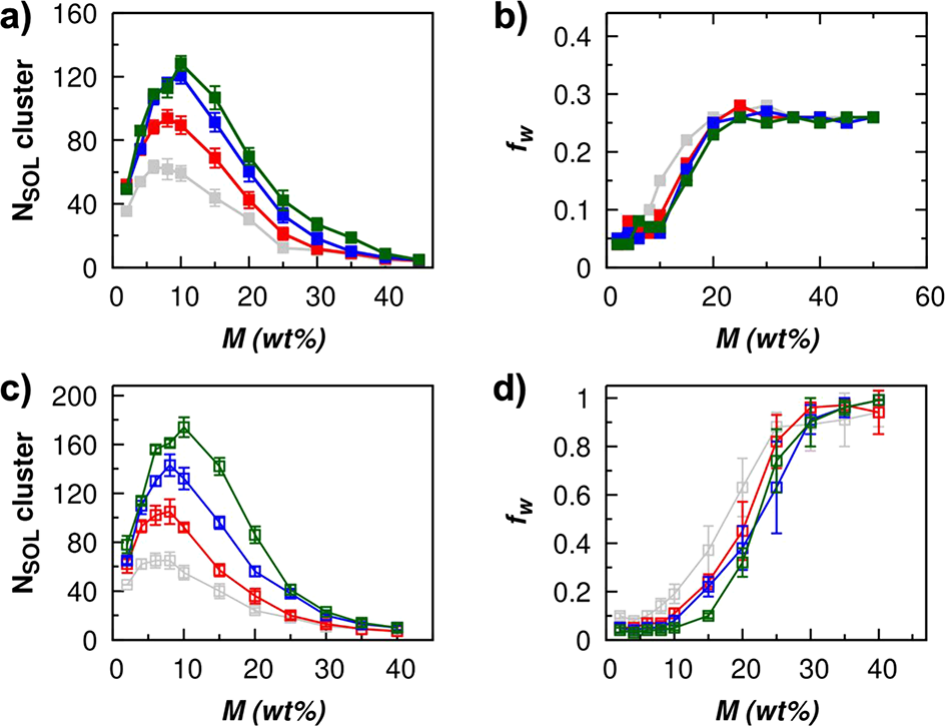
Idealized model: (a) Number of water clusters (*N*_SOL_) and (b) fraction of largest water cluster size (*f*_w_) found in the membrane with *H* = 1.0 nm. Disordered model: (c) Number of water clusters (*N*_SOL_) and (d) fraction of largest water cluster size (*f*_w_) found in the membrane with *H* = 1.0 nm. The *f*_w_ approaching 1 indicates high pore interconnectivity within the membrane. Symbol definitions: gray filled squares, *i*0QGO; gray open squares, *d*0QGO; red filled squares, *i*10QGO; red open squares, *d*10QGO; blue filled squares, *i*20QGO; blue open squares, *d*20QGO; green filled squares, *i*30QGO; green open squares, *d*30QGO.

The adsorption mechanism of water within the membrane channels is analyzed by looking at the favorable sites of adsorption and the evolution of the water cluster number and size with loading, *M*. We carry out the cluster analysis on all our GO membrane models with a *H* = 1.0 nm using an in-house code by Williams et al.^59^ A water cluster is defined as 2 or more water molecules that are connected within an intermolecular distance of 0.36 nm. The largest water cluster size in each GO model can be translated as the fraction, *f*_w_, of the total number of water molecules adsorbed. The value of *f*_w_ ranges from 0 to 1, where *f*_w_ = 1 means that all of the water molecules within the membrane are in one single cluster and indicates full interconnectivity of hydration channels in the membrane. These two quantities as a function of *M* are shown in Figure 6. Note that the range of *M* in the idealized and disordered model is different due to the different membrane capacities as seen in Figure 3a.

As mentioned above and shown in our previous study,^46^ the most favorable sites for adsorption in the charged GO model are the ionized OH groups and this is true in both idealized and disordered models (SI Figure S1). At a low RH, most water molecules are adsorbed at the ionized OH functional groups. At this low-humidity value, the number of water clusters increases as the water molecules filling the membrane are adsorbed at different favorable sites. With increasing humidity and loading, the favorable sites become fully saturated. At this point, the water molecules within the membrane channel start to act as new adsorption sites for new water molecules to be adsorbed, and the adsorption is driven by water–water interactions. Thus, the number of water clusters stops increasing as the currently available water clusters grow and start to coalesce with the neighboring clusters. The highest number of water clusters that can be formed within each membrane model is limited by the number of available ionized OH functional groups. The trend is the same for both idealized and disordered models where the number of water clusters formed is higher in membranes with a higher amount of ionized OH functional groups. The merging of the smaller water clusters with larger ones is indicated by the decrease in the overall number of clusters and the increase in their size. Figure 6 shows that the coalescence of the clusters starts when the membranes have adsorbed around *M* ≈ 10% independently of their topology. In the idealized GO membrane model, the merging continues until all of the clusters in each channel are connected to form water layers, which happens for *M* ≳ 25 wt % (Figure 6b). In this case, since the GO flakes are continuous through the periodic boundary of the simulation box, no interconnectivity between the channels can be achieved, and the maximum value of *f*_w_ is 0.25 (a single water cluster for each channel). In the disordered model, the pores between each layer are highly interconnected, with a *f*_w_ value approaching 1 as *M* increases. The *f*_w_ of the disordered models prove the presence of high pore interconnectivity within the GO membrane that is attributed to the fast permeation of water.^13,14^ Thus, our simulations indicate that water molecules are initially adsorbed in the most hydrophilic regions of the membrane and then from clusters that grow to fill the less hydrophilic regions. This phenomenon occurs at every channel of the membrane simultaneously with similar probability due to the presence of the favorable sites (deprotonated OH) on each side of the GO flakes and is independent of the *H*. This adsorption mechanism provides a mechanistic description that agrees with the theory of the intrastratification (*within one channel*) inferred by observing the gradual shift of X-ray diffraction peaks as RH increases.^13,17^

### Thermodynamic Stability of GO Membrane

**Figure 7:**
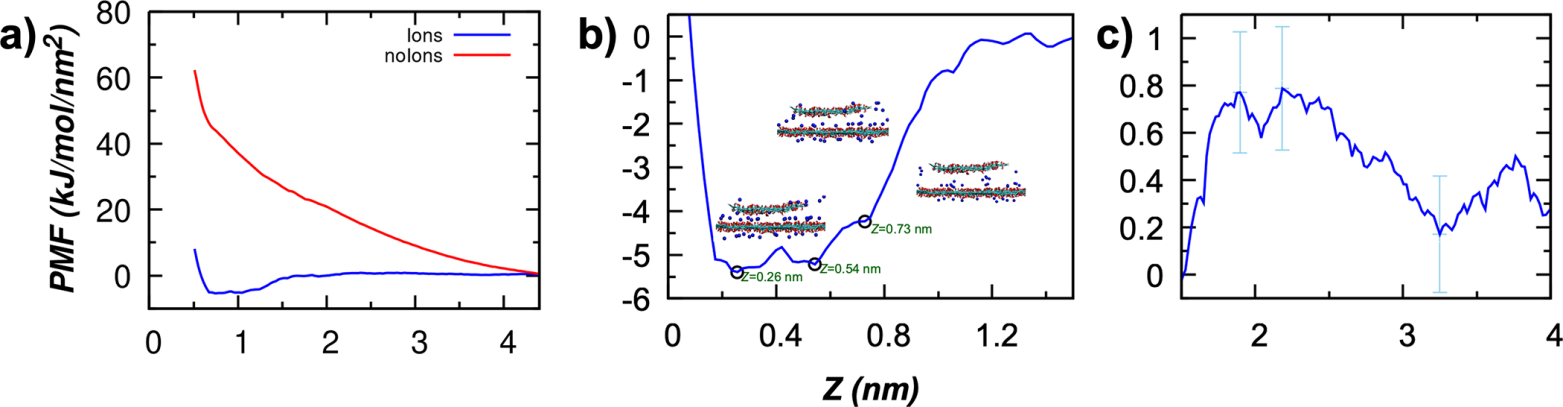
(a) Potential mean force (PMF) of charged GO flakes with (blue) and without (red) counterions. (b) PMF profile with counterions zoomed-in between *Z* = 0.1 nm and *Z* = 1.2 nm with minima found at 0.26, 0.54, and 0.73 nm. Inset of panel b: snapshots of GO flakes with counterions at the corresponding minimum location. (c) PMF profile with counterions zoomed-in for *Z* > 1.5 nm that shows an energy barrier of 0.8 (kJ/mol)/nm^2^. The histogram sampling is provided in SI Figure S6.

In order to assess the thermodynamic stability and further elucidate the contribution to swelling due to electrostatic repulsion, potentials of mean force (PMF) were calculated between two GO sheets corresponding to the 20QGO membrane models (20% hydroxide ionization). The PMFs, shown in Figure 7, were calculated separately for a simulation box with and without Na^+^ counterions. Figure 7a shows that the presence of counterions is crucial for predicting the correct shape of the PMF profile. With counterions, the PMF plateaus at large distances (>3.5 nm), given the long-range repulsive interactions, are sufficiently weak that the slope is not visible beyond the statistical noise. The PMF is weakly repulsive at intermediate distances (1.5–3.5 nm, Figure 7c) due to the screened electrostatic interaction, resulting in a free energy barrier of 0.8 (kJ/mol)/nm^2^, and then it becomes attractive at short distances with a broad free energy minimum from 0.1 to 1.2 nm with a well depth of −5.4 (kJ/mol)/nm^2^. This result is in qualitative agreement with reports elsewhere^28,60^ and consistent with the experimental observation of swelling up to 1.2 nm^27^ beyond which the PMF slope increases dramatically. An examination of this large energy minimum reveals three weakly identifiable features (Figure 7b), with one corresponding to interlayer distances consistent with a sandwiched monolayer (*H* = 0.73 nm) of water.^46^ Although a mean-field approach, such as that based on DLVO theory and previously reported for GO by Gudarzi,^47^ can be used for colloidal systems, this approach is not suitable for investigating the stability of GO membranes in the range of interlayer distances relevant to the swelling process (*H* = 0.5–1.4 nm), which requires consideration of the complex competing interactions occurring at the atomistic length scale. For example, Figure 7b shows that counterions prefer to reside at the GO–water interface and particularly within the channel, bridging between the two adjacent GO sheets.^27,41^

## Conclusions

In this work, we have shown that GO membranes have a dynamic morphology that depends on the relative humidity to which they are exposed to. Systematically comparing the amount of water adsorbed in different GO model membranes, we were able to link experimental adsorption data with an atomistic description of the membrane morphology and surface chemistry and show how these evolve as the membrane is exposed to increasing humid environments. Our simulations show that, at the initial stage of membrane preparation (after drying), the membrane is in a neutral state with a disordered morphology that includes lamellas and irregular pores. As water is adsorbed, the surface chemistry of the GO flakes starts to change as the functional groups deprotonate reacting with water in the pores, resulting in a net negative surface charge within the membrane. As the amount of water adsorbed increases with RH, the GO membrane acquires a surface charge due to the deprotonation of the OH functional groups. The adsorbed water molecules form clusters that grow and coalesce with neighboring clusters that finally form intercalated water layers. The solvation pressure from the confined water layers and the strong electrostatic repulsion from surface charges lead to structural changes of the GO membrane as the membrane swells further. The disordered morphology of the GO membrane observed after its preparation and up to RH = 80%, changes to more ordered and idealized lamellar-like channels as the membrane swells with increasing humidity to accommodate more water layers. Our result shows that at three different RHs of 10, 80, and 98% investigated here, GO membranes are best represented by three different models: a neutral highly disordered model (*d*0QGO), a highly charged disordered model (*d*20QGO), and an ordered slit-like (idealized) highly charged model (*i*20QGO), respectively. These models describe the dynamic properties of the surface chemistry and topology of the GO membrane with increasing RH going from uncharged/disordered to charged/disordered and finally charged/idealized.

At RH = 80%, two disordered models (*d*20QGO and *d*30QGO) have a comparable result in depicting the surface chemistry of the GO membrane. The *d*30QGO is a hypothetical value of surface charge that is unrealistic and out of range of the surface charge values observed in experimental studies^36,47^ and should be ruled out. This result further proves that, as the RH approaches the saturated vapor liquid equilibrium, the topology of the membrane is the main controlling factor in determining its capacity.

The result from our simulation study shows that GO membranes are indeed flexible with dynamic morphology and surface chemistry at different water contents and that an appropriate molecular model must be used depending on the conditions of the environment.

## Methodology

### Calibration Curve of Chemical Potential and Relative Humidity

In this work we performed a series of GCMC simulations with MCCCS Towhee version 8.2^61^ to characterize the water adsorption mechanism and membrane swelling as a function of the ambient RH. To carry out these simulations, it is vital to have a water model that correctly reproduces the water properties at the saturation point and at any other partial pressure values. Thus, we first obtained a calibration curve to find the relationship between the value of the vapor pressure, p* (controllable in the experimental setup), with the chemical potential, μ (which can be fixed for the GCMC adsorption simulations). Such a correlation between p* and μ will allow us to compare our simulation adsorption results directly with the experimental data. Following our previous work for the simulations, we use the TIP3P-EW water model^62^ since a three sites water model is computationally cheaper in a serial Monte Carlo (MC) simulation compared to a four sites water model. Besides, the TIP3P-EW water model has been optimized for Ewald summation, which is important in our simulation that includes ions, and because it is able to reproduce the bulk water density of 997 kg/m^3^. The first step of constructing this correlation was to determine the values of the saturation chemical potential, μ_sat_, and pressure for the TIP3P-EW water model at 300 K. To do that a Gibbs ensemble Monte Carlo simulation consisting of one liquid box in equilibrium with one vapor box of water was used. The Gibbs ensemble simulation was carried out at constant temperature, volume, and total number of water molecules inside both simulation boxes. The initial number of water molecules was 900 in the liquid box and 50 in the vapor box. The dimensions of the simulation boxes were 3 × 3 × 3 nm^3^ for the liquid box and 37 × 37 × 37 nm^3^ for the vapor box, leading to initial densities of 998 and 0.03 kg/m^3^ for the liquid and vapor phases, respectively. The water molecules within both boxes were allowed to rotate and translate within each box with a 45% probability, while the molecule exchange moving between boxes had a probability of 4%. A 1% aggregation volume bias move^63^ to efficiently prevent aggregation of water molecules in the vapor phase and a 4% configurational bias move to regrow water molecules in each simulation box were also applied. The two simulation boxes also had a 1% probability of exchanging the volume between them while keeping the overall volume of the system constant. The vdW interactions between atoms were calculated using a shifted Lennard-Jones (LJ) potential with a cutoff at 1.2 nm, and the cross-species LJ parameters were calculated using Lorentz–Berthelot mixing rules. The electrostatic interactions were calculated using the Ewald summation with the real space cutoff set at 1.2 nm. The simulation box was periodic in all directions. The system was equilibrated until the density, number of water molecules, and pressure within the liquid and vapor box reached a steady state. After equilibration, a production simulation of 25 × 10^6^ MC steps was carried out for data collection. Block average output was collected every 5.0 × 10^5^ MC steps to calculate the pressure of the system to obtained *p*_sat_. The total chemical potential value was calculated using the insertion Rosenbluth weight (eq 3) each time a multibox molecule swap move was attempted.
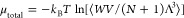
3where *k*_B_ is the Boltzmann constant, *T* is temperature, *W* is the Rosenbluth weight, *V* is the volume of simulation box, *N* is the number of water molecule, Λ is the thermal de Broglie wavelength of water molecule, and the angular brackets denote ensemble averaging. The test particle insertion method is known to fail for dense fluids because of strong overlaps with the test particle.^64^ Thus, only values of μ_sat_ and *p*_sat_ calculated from the Gibb’s ensemble simulation of vapor box were used to calculate the saturation properties of the TIP3P-EW water. The value of μ_sat_ obtained from Gibbs ensemble vapor box was then validated for the saturated liquid water with a GCMC simulation performed on a liquid water box. The GCMC simulation was able to reproduce the correct density of liquid water for TIP3P-EW (997 kg/m^3^) at 300 K.

Once the saturation point properties, μ_sat_ and *p*_sat_ were established, the two values were used as reference points to generate a set of values of chemical potential corresponding to lower p*. Thus, a series of GCMC simulations with μ values lower than μ_sat_ were carried out for vapor boxes of dimension of 85 × 85 × 85 nm^3^ with 500 initial water molecules to calculate the corresponding p* at 300 K. The MC moves considered in these GCMC simulations were insertion/deletion, translation, and rotations. In the GCMC simulation, the insertion/deletion moves were given a high priority with 50% probability to speed up the simulation, while the translation and rotation moves each have 25% probability. The system was equilibrated until the number of water molecules in the simulation box reached a steady state. Then, production runs were carried out for 15 × 10^6^ MC steps to calculate p*. The value of p* calculated at each μ value was then translated into RH using eq 4.
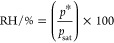
4where *p*_sat_ is the value of saturated vapor pressure calculated from the Gibbs ensemble simulation. The values of μ and the corresponding RH were then fitted to create a calibration curve. The calibration curve was then used as a guide to interpolate the value of μ needed for the GCMC adsorption simulations at specific RH values guided by the experimental literature.

**Figure 8:**
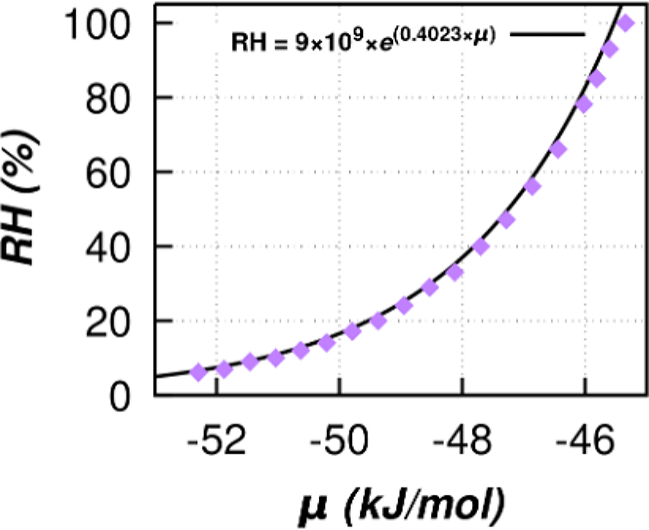
Calibration curve between relative humidity (RH) and the chemical potential of water (μ). Purple diamond is the calculated value of μ from simulation, while the black dashed line is the best fit line. The equation of the best fit line (eq 5) is in the top center.

The value of μ_sat_ calculated from the Gibbs ensemble simulations is −45.34 kJ/mol with a saturation pressure, *p*_sat_, of 38.2 mbar. The calibration curve linking μ to RH is plotted and is shown in Figure 8. A best fit line applied on the series of data sets gives an exponential correlation shown in eq 5.
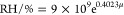
5

The fitting parameters are within 3% error from thermodynamically derived μ in the form μ = μ_sat_ + *k*_B_*T* ln(RH). Equation 5 is used to translate the input value of μ in the GCMC adsorption simulations to RH values to enable direct comparison of our simulated results with the available experimental data.

### Graphene Oxide Membrane Preparation (Disordered/Idealized)

Two types of GO membrane models were built to study the effect of the membrane topology on the water adsorption properties and the swelling mechanism. These two models differ regarding the degree of disorder in their structure with one (the “idealized model”) having all of the GO flakes almost parallel to each other and the other one (the “disordered model”) resembling the amorphous structure of experimental membranes (see Figure 1). The value of *H* was decided based on a literature experimental result by Liu et al. correlating *H* with RH^6^ that shows a gradual increase of *H* from 0.8 to 1.0 nm for RH of 10 to 98%, respectively. We therefore built our GO model with *H* of 0.8, 0.9, and 1.0 nm and used them in GCMC simulations performed at RHs of 10, 80, and 98%, respectively. We also fabricated a fourth GO model with *H* of 1.3 nm which is the *H* observed experimentally when GO membranes are immersed in liquid water. As GO membranes acquire a surface charge when exposed to a humid environment due to the reaction between functional groups donating their proton to water molecules to form hydronium ions,^38,48^ in conjunction with the topology effect, we also investigate the effect of surface charge. The surface charges assigned to the GO flake were calculated based on the number of deprotonated hydroxyl functional groups (OH). We model GO membranes with 0, 10, 20, and 30% of the OH groups being deprotonated. The 10 and 20% deprotonation values were within the range of surface charge values reported in experimental studies,^36,47^ while the 30% deprotonation is included for comparison purposes. The 0% deprotonation is representative of the typical neutral model of GO membrane that is widely used in computer simulation studies. Thus, overall, we simulated 60 different GO membrane models which we label as *jk*-GO where the value of *j* refers to the model topology type with *i* indicating the idealized topology and *d* the disordered one, while *k* refers to the percentage of deprotonation of each flake which can be neutral with 0% (*k* = 0Q) or can have a net charge of 10% (*k* = 10Q), 20% (*k* = 20Q), or 30% (*k* = 30Q) weight percentage. Thus, the models labeled as *i*0QGO, *i*10QGO, *i*20QGO, and *i*30QGO are the GO membrane models with idealized topology and with 0 (i.e., neutral), 10, 20, and 30% surface charges, respectively, while the *d*0QGO, *d*10QGO, *d*20QGO and *d*30QGO models are the equivalent with disordered topology.

The fabrication method of the membrane is different, depending on the type of model produced. For the idealized GO membrane model, we followed the procedure used in our previous work,^46^ while for the disordered GO membrane model we followed the procedure reported by Williams et al.^43^ with an additional step of removing a vacuum gap using a pressure of 100 bar in the membrane with a net negative surface charge. During this extra step of MD simulation, a Nosé–Hoover thermostat and a Parrinello–Rahman barostat is used. Each GO flake originated from a graphene sheet generated from VMD version 1.9.2^65^ and then oxidized with functional groups using the in-house code by Williams et al.^43^ where all the atoms on GO flakes are parametrized with CHARMM potential.^66^ The oxygenated functional groups used to functionalize the graphene flake were hydroxyl (OH) and epoxide (OE) in a 1:1 ratio. These were randomly distributed with a total oxygen weight percentage of 30%. In the case of GO with net surface charge, some of the OH groups are deprotonated. The oxidation process was repeated four times for each GO flake to produce four randomly oxidized GO flakes with the same surface chemistry. Sodium ions, Na^+^, with LJ parameters from Joung and Cheatham,^67^ were randomly inserted to neutralize the simulation box with net negative surface charge (i.e., there are no ions in the neutral models). Molecular dynamics (MD) simulations, performed with GROMACS version 2021.2,^68^ were used to relax the GO flakes for the idealized model and to carry out the procedure of fabricating disordered GO models.

In the idealized model, the size of *H* is measured as the distance between the centers of mass between opposing GO flakes, while, in the disordered model, the average size of *H* is determined by using eq 6;
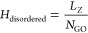
6where *L*_*Z*_ is the length of the simulation box in the *Z* direction and *N*_GO_ is the number of GO flakes in the membrane which is 4. To improve the statistics, we built three models for each *H* of the disordered model for the GCMC adsorption simulations. Figure 1 shows the final structure of the disordered and idealized GO membrane model for *H* = 1.0 nm and 10% ionization of OH functional groups as example (*d*10QGO and *i*10QGO). There are no carboxylic functional groups added in our GO model even if it is widely reported in literature to be present at the edges of GO flakes and influence the permeation of water and selectivity toward ions.^20,69^ Since our idealized GO model is continuous periodically and has no edges, we only use hydrogen atoms to terminate the edges of the disordered flake to ensure a similar comparison. This is reasonable in our case since we are focusing on the swelling of the GO membrane and simulating our system with GCMC simulations that do not consider any dynamic properties. Besides, the size of our GO flake is very small compared to experimental GO sheets and including carboxylic groups at the edges would overshadow the effect of surface charges, which are more prominent to swelling.

### Water Adsorption in Graphene Oxide Membrane Simulation

The GO membrane models were then used to carry out GCMC simulations to obtain the amount of water adsorbed under various humidity conditions and to reproduce experimental data. The values of *H* and the corresponding RH for the GCMC simulation were decided based on the experimental isotherm of adsorption reported by Liu et al. that relates *H* with RH.^6^ The list of GO membrane models and their surface charge values are shown in Table 2.

**Table 2:** List of the GO Model Used in This Study and the Value of Surface Charge from Deprotonation of OH Functional Groups^a^

model name	*σ*(mC/m^2^)
*i*0QGO	0.00
*i*10QGO	–49.66
*i*20QGO	–101.58
*i*30QGO	–148.98
*d*0QGO	0.00
*d*10QGO	–54.40
*d*20QGO	–108.80
*d*30QGO	–155.20

a Each charged model is fabricated for four *H* values of 0.8, 0.9, 1.0, and 1.3 nm, while the neutral model is fabricated in three *H* values of 0.8, 0.9, and 1.0 nm.

The adsorption simulations were started with a dry GO membrane that was placed in equilibrium with a hypothetical water vapor bath at the chosen RH. During the adsorption simulations, the whole GO membrane was kept rigid, and MC moves were applied only to the water molecules and Na^+^ ions. Water molecules undergo center of mass translation, rotation, and insertion/deletion moves, while the Na^+^ ions only undergo center of mass translation moves. The vdW and electrostatic interactions were treated in the same way as described for the pure water Gibbs ensemble simulations. The simulation box was equilibrated until the number of water molecules adsorbed within the GO membrane reached a steady state. Production runs were then carried out for 4 × 10^6^ MC steps for data collection to calculate the amount of water adsorbed, *M*, using eq 2. In the disordered GO membrane model, the number of water molecules adsorbed was averaged over the three models with the same *H*.

### Calculation of PMFs

PMFs were calculated using an umbrella sampling approach^70,71^ and a pair of GO sheets in a simulation box with dimensions of 5.2 × 5.1 × 10 nm^3^. Both GO flakes have a surface charge equivalent to the 20% ionization hydroxyl functional groups, i.e., 20QGO membrane models. This surface charge was chosen based on the study by Konkena and Vasudevan,^36^ who calculated the stability of GO flakes dispersion at different pH values. The *xy*-dimensions of the first sheet were chosen to match those of the simulation box and placed near the base of the simulation box (*z* = 0.5 nm). The coordinates of this sheet were frozen throughout the simulations. A second, fully flexible GO sheet with dimensions of 3.2 × 3.4 nm^2^ was initially placed at *z* = 5 nm. Sodium counterions were randomly added to the simulation box to satisfy the electroneutrality requirement. Finally, the box was solvated with water. The initial coordinates for the umbrella sampling procedure were generated by pulling a randomly selected carbon atom in the flexible GO flake from its initial position toward the frozen GO sheet using a harmonic potential with a force constant of 1 × 10^6^ (kJ/mol)/nm^2^. This choice of reaction coordinate means that the flexible GO flake can freely adopt any orientation relative to the frozen GO sheet. Snapshots were chosen for 40 separate simulations with *z*-coordinates ranging from 0.5 to 4.4 nm and an interval of 0.1 nm. In each umbrella sampling simulation, the *z*-coordinate of the randomly selected atom was held constant using a biasing force constant of 1 × 10^4^ (kJ/mol)/nm^2^. Biased coordinates and forces obtained at the various *z*-coordinates were unbiased and recombined by using the weighted histogram analysis method (WHAM). PMFs were calculated in both the presence and absence of counterions. The simulation setup is shown in Figure S5. The force-field parameters for GO flakes, water molecules, and sodium ions are the same as described above in the MD simulation setup of the membrane fabrication. Each simulation is carried out in an *NVT* ensemble for 5 ns.

### Experimental Determination of Water Adsorption Isotherm in Graphene Oxide Membrane

A gravimetric technique was employed to measure adsorption isotherms of the GO membrane samples using an Intelligent Gravimetric Analyzer, IGA-003. The IGA-003 is equipped with an ultrasensitive microbalance of resolution 0.2 μg mounted in a high-precision temperature-controlled heat sink. About 5 mg of GO membrane was weighed for each measurement before outgassing at 50 °C and 100 mbar pressure under a dry N_2_ flow of 100 mL/min until the weight of the samples reached a stable minimum.

Adsorption isotherms were measured in dynamic mode at 1 bar pressure under a gas flow of 100 mL/min at 27 °C. The sample temperature was controlled precisely by an external water jacket connected to a constant-temperature bath. To control the RH, solvent vapor saturated nitrogen gas was mixed with dry N_2_ at the appropriate proportion using mass flow controllers before being introduced into the sample chamber. The end point for each step was measured by a real-time processor considering a 99.9% relaxation.

## Supporting Information Available

Radial distribution function plots; pore size distributions; density profiles; tables on adsorbed water amounts and on adsorbed water cluster distributions; snapshots of GO flakes and counterions positions from PMF simulation; PMF simulation setup; WHAM sampling histogram (PDF)
