# Supplementary material for: Decoding the Interplay between Topology and Surface Charge in Graphene Oxide Membranes During Humidity Induced Swelling
Source: ACS Nano. 2023 Nov 2;17(21):21923–34. doi: 10.1021/acsnano.3c08260 (PMC10655246; doi:10.1021/acsnano.3c08260)
Supplement: Supplementary file 1 — nn3c08260_si_001.pdf [file nn3c08260_si_001.pdf]

Decoding The Interplay Between Topology and Surface Charge in Graphene Oxide Membranes During Humidity Induced Swelling.

Supporting Information

Mohd Rafie bin Shaharudin[1], Christopher D. Williams[2], Amritroop Achari[1,3], Rahul R. Nair[1,3], Paola Carbone[1]*.*

*mohdrafiebin.shaharudin@manchester.ac.uk

*paola.carbone@manchester.ac.uk

[1]Department of Chemical Engineering, School of Engineering, The University of Manchester, Booth Street East, M13 9PL, Manchester, United Kingdom

[2]Division of Pharmacy and Optometry, School of Health and Sciences, The University of Manchester, Oxford Road, M13 9PT, Manchester, United Kingdom

[3]National Graphene Institute, The University of Manchester, Booth Street East, M13 9PL, Manchester, United Kingdom

To investigate the chances of hydrogen bond forming, we calculate the radial distribution function (RDF) between ionised hydroxyl (qOH) functional groups and the hydrogen atom of water molecules (HW). The first peak of the qOH-HW RDF is at 0.175 nm which is within the range of geometrical definition of hydrogen bond. The RDFs of other oxygenated functional groups (epoxide, OE and unionised hydroxyl, OH) do not show a strong first peak suggesting that the most favourable site for adsorption is the ionised hydroxyl functional group.

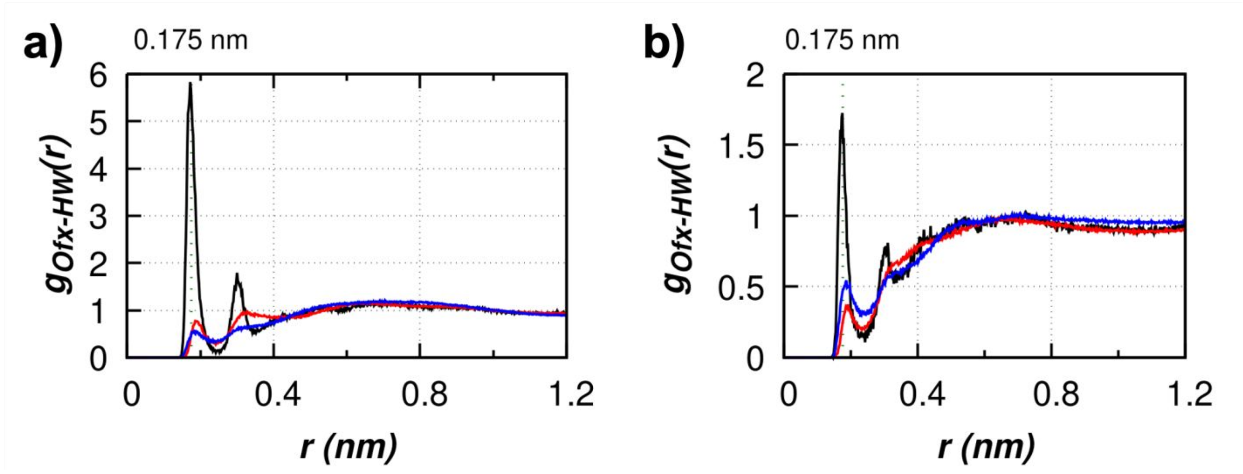

Figure S1. Radial distribution function (RDF) between qOH-HW (black), OH-HW (blue) and OE-HW (red) of a) idealised model) and b) disordered model at low RH.

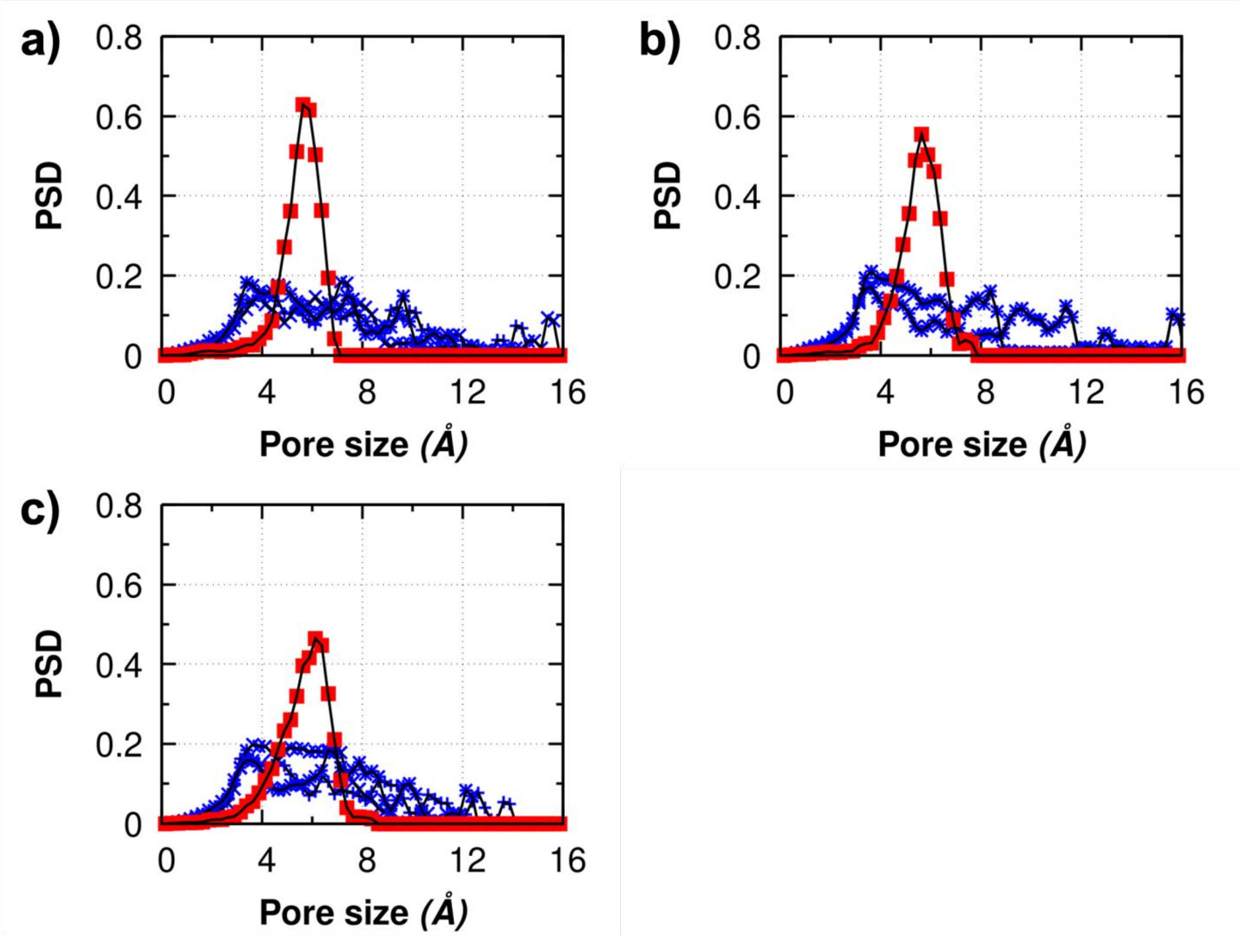

Figure S2. Pore size distribution of the idealised (■) and disordered model 1 (+), model 2 (×), and model 3 (∗) GO membranes of a) 10QGO, b) 20QGO, and c) 30QGO at $H = 1.0$ nm.

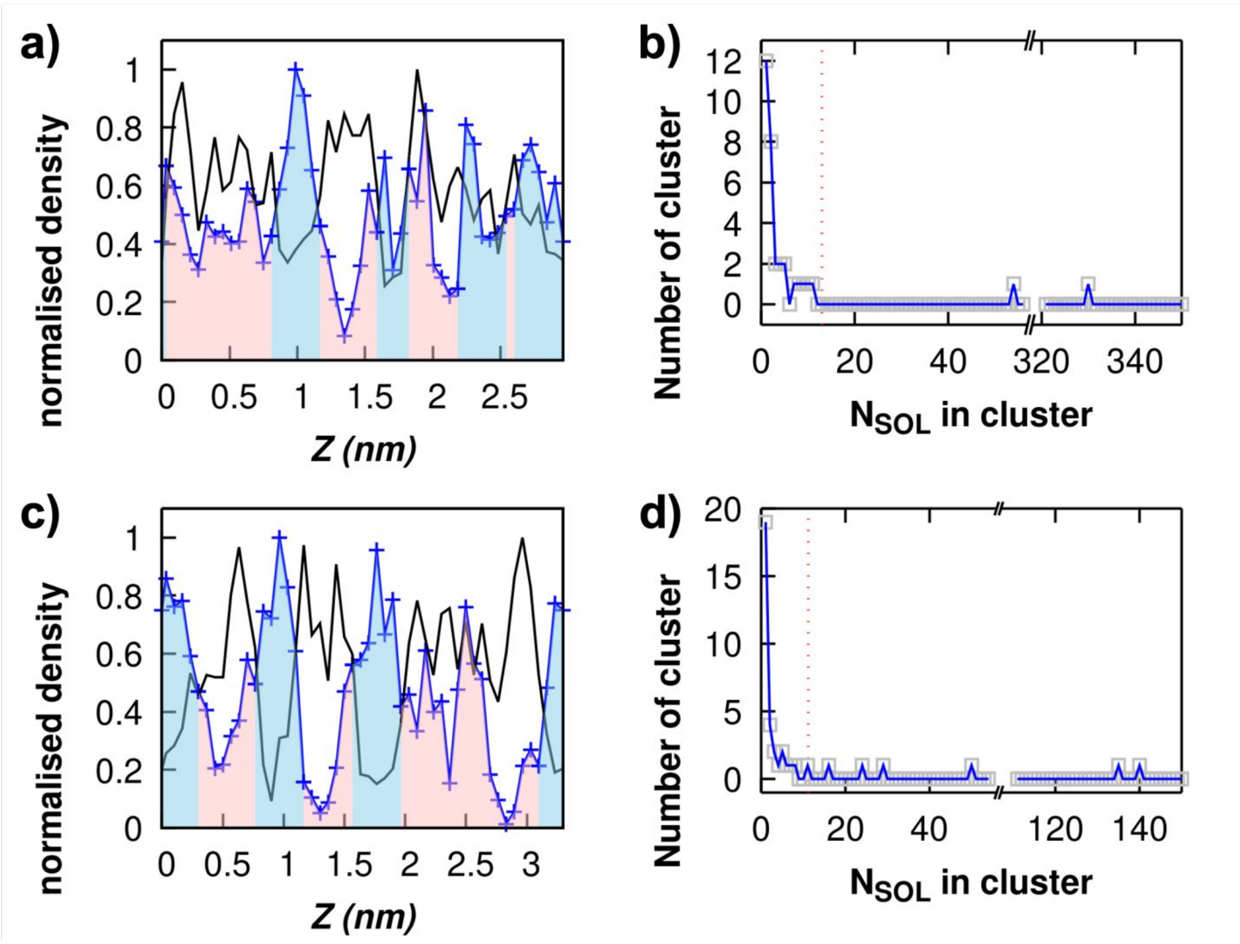

Figure S3. Density profile in Z direction for water and GO flakes of a) *d*0QGO model 2 and c) *d*0QGO model 3 and the water cluster distribution of b) *d*0QGO model 2 and d) *d*0QGO model at *H* = 0.8 nm.

Table S1. The amount of water adsorbed, *M*, in GO membrane model at RH = 100% and *H* = 1.3 nm for 10QGO, 20QGO and 30QGO.

	**GO membrane**	**RH (%)**	***H* (nm)**
		**100**	**1.3**
***M* (wt%)**	*d*10QGO	65.31±0.07	
	*d*20QGO	64.42±0.11	
	*d*30QGO	64.39±0.12	
	*i*10QGO	76.26±0.06	
	*i*20QGO	79.12±0.03	
	*i*30QGO	80.68±0.03	

Table S2. The amount of water adsorbed, *M*, in isolated pores of *d*0QGO at RH = 10% across all three models and their average with different limit of cluster size.

***d*0QGO**	**Isolated *M* (wt%)**				
	Mean cluster as limit	Cluster limit = 10	Cluster limit = 50	Cluster limit = 100	Total *M*
**Model 1**	3.61	3.10	6.55	6.55	12.24
**Model 2**	2.48	1.95	2.48	3.87	12.31

**Model 3**	2.02	1.74	3.79	5.07	12.11
**Average**	2.70	2.26	4.27	5.16	12.22

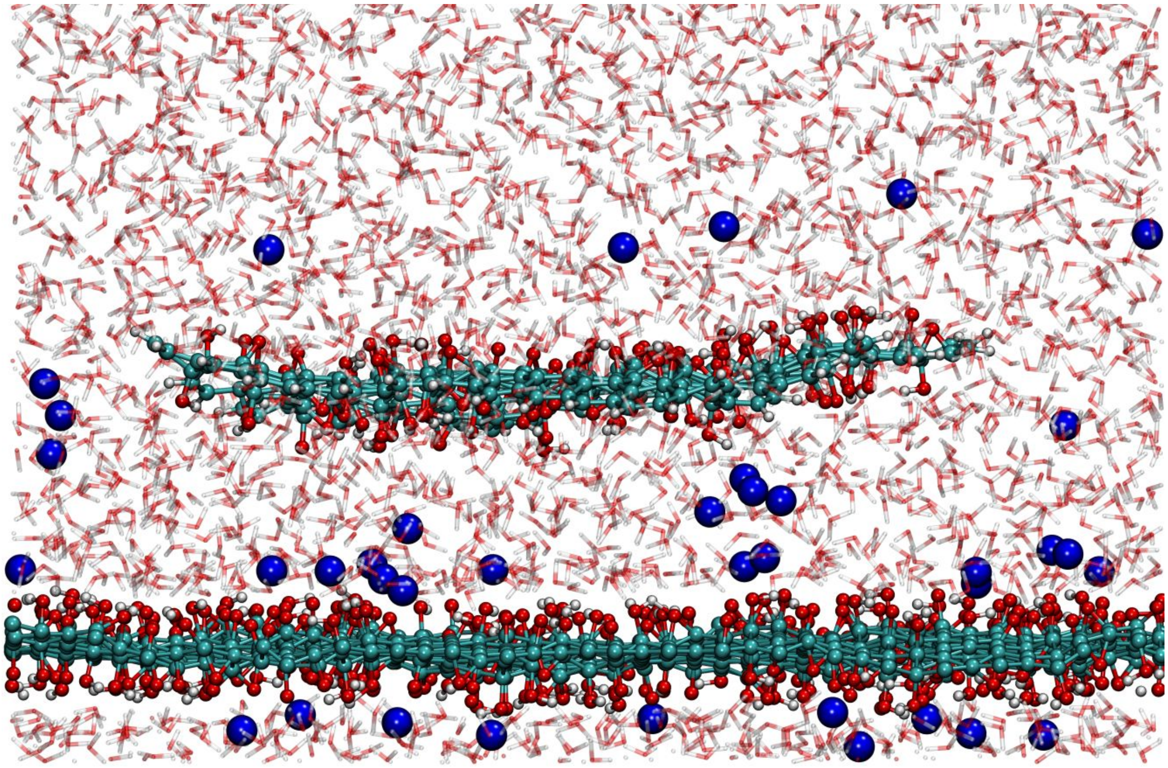

Figure S4. The snapshot from PMF simulation shows that the counter ions prefer to stay in between the channel between two GO flakes. Cyan: Carbon atom, white: Hydrogen atom, red: Oxygen atom, and blue: Sodium ion.

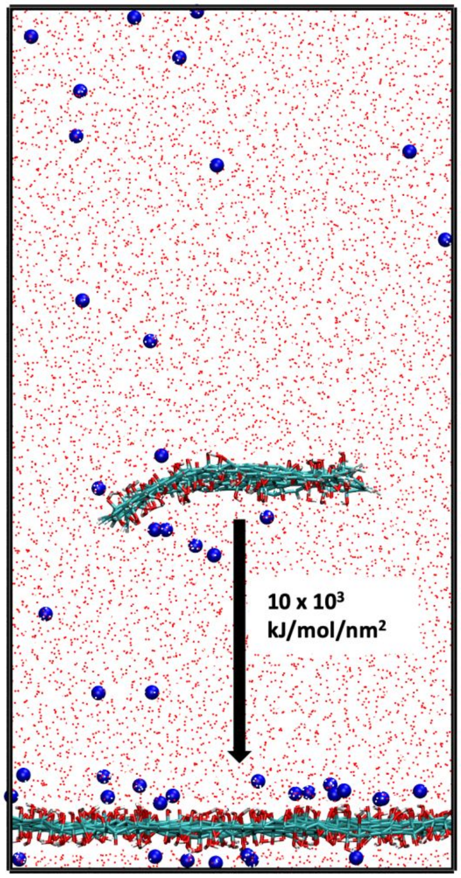

Figure S5. The simulation set up for PMF calculation. The smaller GO flakes with pulling force of 1×10^4 kJ/mol/nm^2 in Z direction towards the frozen larger GO flake have different starting point ranging from 0.8 nm to 4.4 nm to sample the force between two GO flakes at various distances for PMF calculation using WHAM. Cyan: GO flakes, blue: Sodium ions, and red: water molecules.

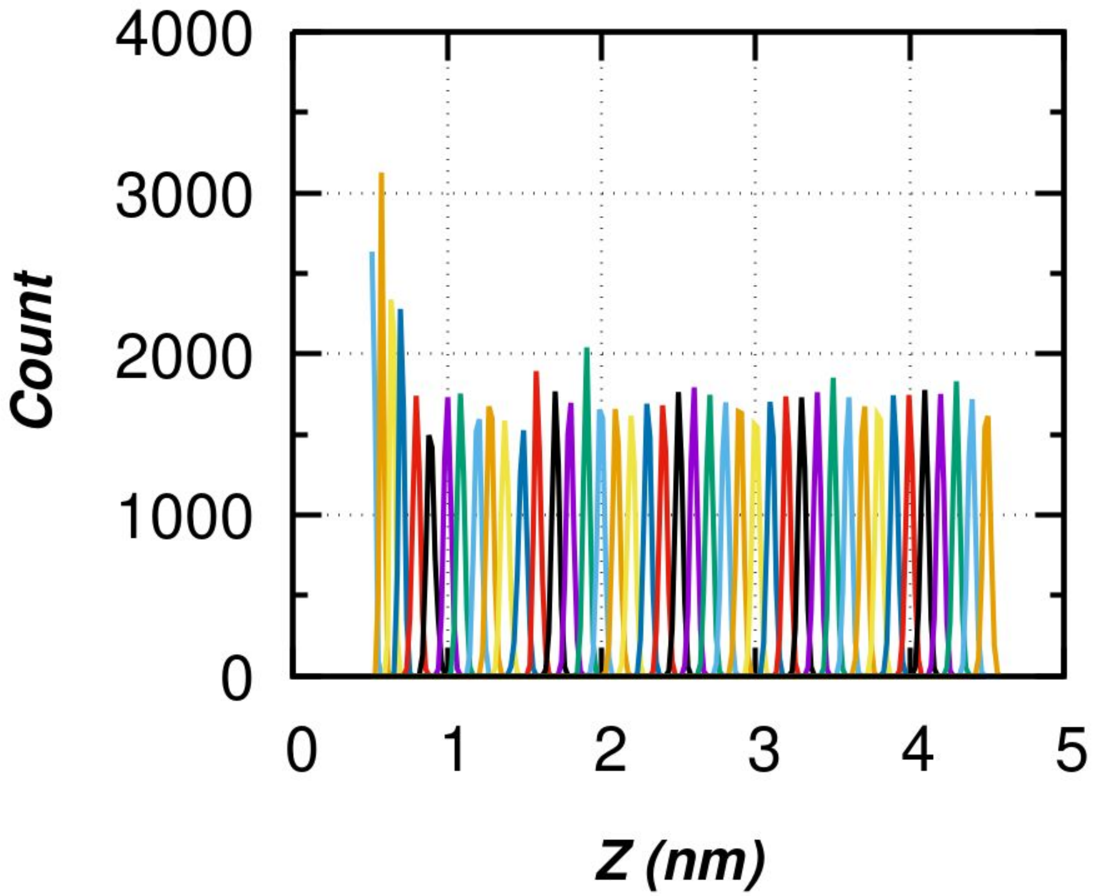

Figure S6. Sampling histogram of PMF calculation using WHAM between two GO flakes with 20% deprotonation hydroxyl functional groups. Sample is taken in Z direction from 0.5 nm to 4.4 nm.
